# A 21-Day of Adjunctive Corticosteroid Use May Not Be Necessary for HIV-1-Infected Pneumocystis Pneumonia with Moderate and Severe Disease

**DOI:** 10.1371/journal.pone.0138926

**Published:** 2015-09-22

**Authors:** Satoshi Shibata, Takeshi Nishijima, Takahiro Aoki, Yoshinari Tanabe, Katsuji Teruya, Yoshimi Kikuchi, Toshiaki Kikuchi, Shinichi Oka, Hiroyuki Gatanaga

**Affiliations:** 1 AIDS Clinical Center, National Center for Global Health and Medicine, Tokyo, Japan; 2 Department of Respiratory Medicine and Infectious Diseases, Niigata University Graduate School of Medical and Dental Sciences, Niigata, Japan; 3 Center for AIDS Research, Kumamoto University, Kumamoto, Japan; Saint Louis University Division of Infectious Diseases and Immunology, UNITED STATES

## Abstract

**Background:**

The current guidelines recommend 21-day adjunctive corticosteroid therapy for HIV-1-infected pneumocystis pneumonia patients (HIV-PCP) with moderate-to-severe disease. Whether shorter adjunctive corticosteroid therapy is feasible in such patients is unknown.

**Methods:**

We conducted a retrospective study to elucidate the proportion of patients with moderate and severe HIV-PCP who required adjunctive corticosteroid therapy for 21 days. The enrollment criteria included HIV-PCP that fulfilled the current criteria for 21-day corticosteroid therapy; PaO_2_ on room air of <70mmHg or A-aDO_2_ ≥35 mmHg.

**Results:**

The median duration of corticosteroid therapy in the 73 study patients was 13 days (IQR 9–21). Adjunctive corticosteroid therapy was effective and discontinued within 10 and 14 days in 30% and 60% of the patients, respectively. Only 9% of the patients with moderate HIV-PCP (n = 22, A-aDO_2_ 35–45 mmHg) received steroids for >14 days, whereas 35% of the patients with severe HIV-PCP (n = 51, A-aDO_2_ ≥45 mmHg) required corticosteroid therapy for ≥21 days. Four (13%) of the severe cases died, whereas no patient with moderate disease died. Among patients with severe HIV-PCP, discontinuation of corticosteroid therapy within 14 days correlated significantly with higher baseline CD4 (p = 0.049).

**Conclusion:**

Shorter adjunctive corticosteroid therapy was clinically effective and adjunctive corticosteroid could be discontinued within 14 days in 60% of moderate-to-severe HIV-PCP and 90% of moderate cases.

## Introduction

Although combination antiretroviral therapy (cART) has substantially improved the prognosis of patients with HIV-1 infection, a large number of patients are still diagnosed with HIV-1 infection at a late stage, often with concurrent opportunistic infections [1]. Pneumocystis pneumonia (PCP) is one of the most common opportunistic infections in patients with HIV-1-infection [2,3], and it is important to provide appropriate management of PCP. The American CDC Guidelines recommend 21 days of adjunctive systemic corticosteroid therapy for moderate-to-severe PCP associated with HIV-1 infection [defined as PCP with room air alveolar-arterial O_2_ gradient (A-aDO_2_) ≥35 mmHg or partial pressure of atrial oxygen (PaO_2_) <70 mmHg] [4]. However, corticosteroid therapy may cause deterioration of cell-mediated immunity and enhance the development of other opportunistic infections, especially in those patients with poor immunity [5–8]. Moreover, although the American CDC guidelines further categorized moderate-to-severe PCP into two categories; severe (defined as A-aDO_2_ ≥45 mm Hg) and moderate (A-aDO_2_ ≥35 mmHg and <45 mmHg, or PaO_2_ <70 mmHg), they recommend the same duration (21 days) of adjunctive steroid therapy for both moderate and severe PCP cases [4]. At this point, whether 21 days of concurrent corticosteroid therapy is necessary for moderate-to-severe PCP in HIV-1-infected patients, especially for moderate cases, is unknown.

Based on above background, this study was designed *1)* to elucidate the proportion of HIV-1-infected patients with moderate and severe PCP who fulfilled the criteria for use of adjunctive corticosteroid for 21 days who actually needed corticosteroid use for 21 days from retrospective chart review, and *2)* to investigate the factors associated with shorter (<21 days) duration of steroid use among such patients. In this retrospective review, the CDC guidelines were not always followed but instead use of adjunctive corticosteroids was modified based on the hospital protocol and treating physician's judgment. Thus, this study discussed what has been done in actual clinical practice and was both a discussion of actual clinical practice and patient outcomes.

## Patients and Methods

### Study design

We performed a single-center retrospective chart review of HIV-1-infected patients using the medical records at the National Center for Global Health and Medicine, Tokyo, Japan [9]. The study population was HIV-1-infected patients, aged 18 years and older, who was diagnosed with PCP between January 2004 and December 2012. The diagnosis of PCP was required to fulfill either one of following two criteria; confirmed PCP based on *1)* history of shortness of breath, dyspnea on exertion, and cough; and *2)* histological or cytological evidence of *Pneumocystis jirovecii* in bronchoalveolar lavage fluid, or probable PCP based on *1)* history of shortness of breath, dyspnea on exertion, and cough and *2)* abnormal CT scan findings compatible with PCP, and *3)* initiation of specific anti-pneumocystis therapy [10]. Both confirmed and probable PCP cases were required to fulfill the criteria for 21-day use of corticosteroids recommended by the American CDC Guidelines; PaO_2_ on room air breathing of <70 mmHg or A-aDO_2_ ≥35 mmHg [4]. The two exclusion criteria were *1)* patients who were already treated with corticosteroids at the time of diagnosis of PCP, and *2)* patients in whom blood gas analysis was not examined. At our clinic, we have a hospital protocol for the prescription of steroid for PCP with moderate to severe disease: start with 80 mg of prednisolone orally for 3 days, and taper to 40 mg for 3 days, and further taper to 20 mg for three days and discontinue corticosteroid, if a clinical course of the patient goes well, based on the results of follow-up blood gas analysis, chest X-ray, the amount of supplemental oxygen, and oxygen saturation on exertion. However, duration and dosing of adjunctive corticosteroids is based on clinical conditions and varies across individuals. Anti-PCP treatment by use of either sulfamethoxazole/trimethoprim, intravenous pentamidine, or atovaquone was continued for at least 21 days.

The study was approved by the Human Research Ethics Committee of National Center for Global Health and Medicine (G-001616-00). All patients included in this study provided written informed consent for their clinical and laboratory data to be used and published for research purposes. The study was conducted according to the principles expressed in the Declaration of Helsinki.

### Measurements

The duration of steroid therapy and total amount of systemic corticosteroid (equivalent to prednisolone) during PCP treatment were collected from the medical charts, in addition to basic demographics (age and sex), date of PCP diagnosis, and cART-experienced or naïve. Laboratory data at PCP diagnosis [CD4 count, HIV-1 viral load, serum β-D glucan, lactate dehydrogenase (LDH), and C-reactive protein] were also collected from the medical charts. Patients were followed up for one year, and prognosis by one year after the diagnosis of PCP was collected.

### Statistical analysis

The variable of primary interest was the duration of systemic corticosteroid therapy during treatment of PCP. The study patients were divided into two groups based on blood gas analysis data; severe disease group (A-aDO_2_ ≥45 mmHg) and moderate disease group (A-aDO_2_ <45 mmHg) based on American CDC Guidelines [4]. Baseline characteristics were compared between the two groups using the Student's *t*-test and χ^2^ test (Fisher’s exact test) for continuous and categorical variables, respectively. To investigate the factors associated with the duration of corticosteroid use among the severe group, the severe group was subdivided into two groups: patients with long corticosteroid use (>14 days) and short corticosteroid use (≤14 days), and baseline characteristics were compared between the two groups. All statistical analyses were performed with The Statistical Package for Social Sciences ver. 21.0 (SPSS, Chicago, IL).

## Results

**Fig 1:**
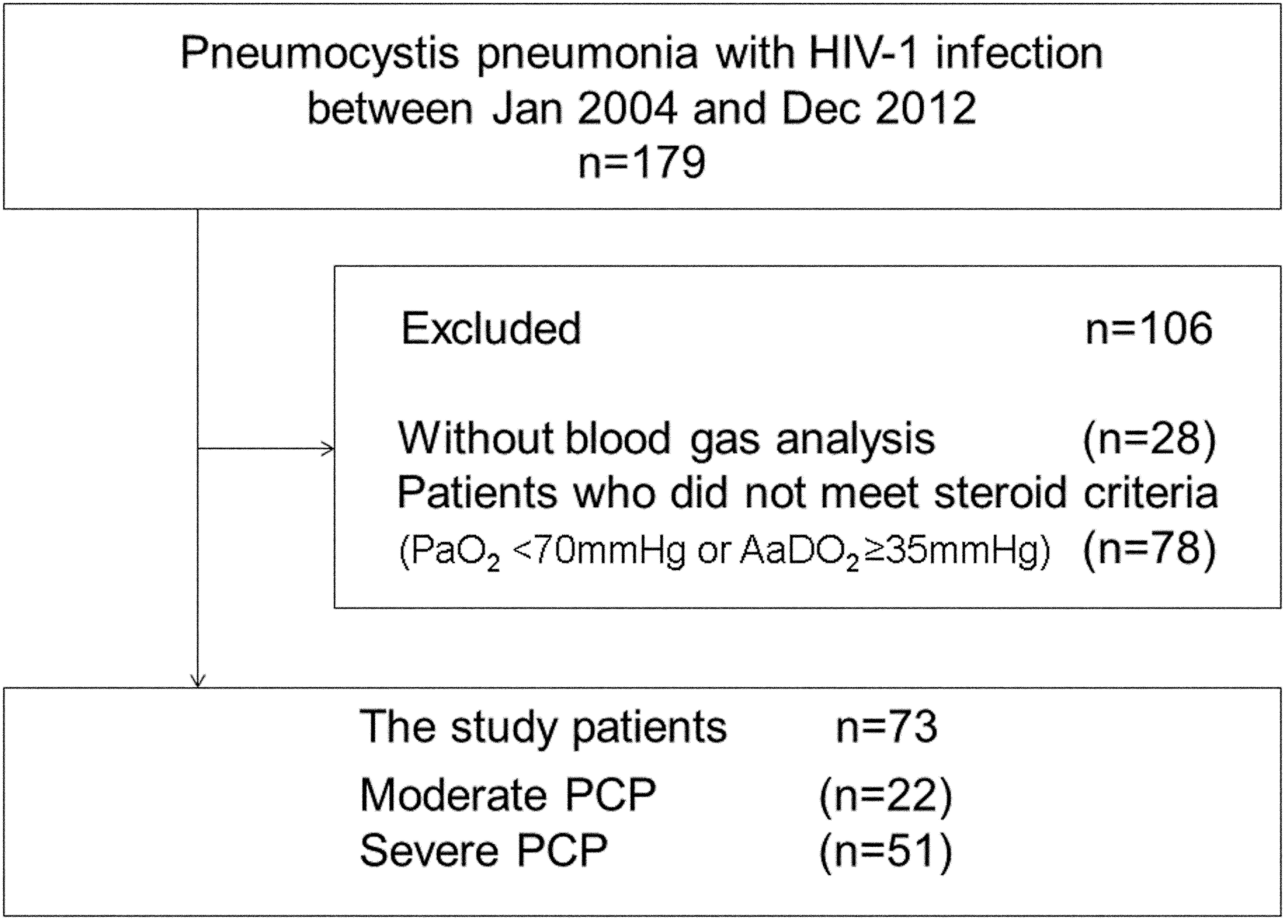
Patient enrollment process.

A total of 179 patients with HIV-1 infection were diagnosed with PCP during the study period. Of these, 28 patients in whom blood gas analysis was not performed were excluded. Of the remaining 151 patients, 73 patients fulfilled the enrollment criteria, and were included in the data analysis (Fig 1). Among the study patients (n = 73), the median age was 39 years and 92% of the enrolled patients were males (Table 1). 40 patients (55%) had histological or cytological evidence of PCP, and were regarded as confirmed PCP cases. 97% were treatment-naïve for HIV infection, and the median CD4 cell count was 35/μL and median HIV-1 load was 5.20 log_10_ copies/mL. The median LDH value was 447 U/L (interquartile range 367–590) and the median β-D glucan was 235 pg/mL (interquartile range 134–962). 75% were infected with other opportunistic infections at the diagnosis of PCP.

**Table 1:** Baseline characteristics of the study patient.

Variables at PCP diagnosis	Total (n = 73)	Severe PCP (A-aDO_2_ ≥45 mmHg) (n = 51)	Moderate PCP (A-aDO_2_ ≥35 and <45 mmHg or PaO_2_ <70 mmHg) (n = 22)	P value
Age (years)^†^	39 (33–48)	39 (34–50)	37 (30–46)	0.16
Males, n (%)	67 (92%)	46 (90%)	21 (95%)	0.45
CD4 count (/μL)^†^	35 (12–66)	35 (12–63)	41 (14–72)	0.69
HIV-1 load (log_10_/mL)^†^	5.20 (4.84–5.73)	5.18 (4.85–5.73)	5.23 (4.78–5.74)	0.79
Histologically or cytologically confirmed PCP, n (%)	40 (55%)	26 (51%)	14 (64%)	0.44
Opportunistic infections other than PCP, n (%)	55 (75%)	40 (78%)	15 (68%)	0.35
Cryptococcosis, n (%)	2 (3%)	2 (4%)	0	
Tuberculosis, n (%)	0	0	0	
CMV end-organ diseases, n (%)	0	0	0	
β-D glucan (pg/mL)^†^ ^¶^	235 (134–962)	224 (137–816)	365 (95–1068)	0.24
PaO_2_ (mmHg)^†^	68 (58–81)	68 (52–94)	67 (65–70)	0.12
AaDO_2_ (mmHg)^†^	56 (42–86)	69 (53–116)	40 (35–40)	<0.001
LDH (U/L)^†^	447 (367–590)	474 (398–685)	379 (327–460)	<0.001
CRP (mg/dl)^†^	5.91 (1.86–9.32)	6.37 (2.39–10.50)	2.79 (1.13–7.82)	0.83
Total corticosteroid dosage (mg) (prednisolone equivalent)^†^	405 (315–1136)	570 (345–4170)	315(184–361)	<0.001
Duration of corticosteroid therapy (days)^†^	13 (9–21)	15 (11–24)	9 (9–13)	<0.001
cART naïve, n (%)	71 (97%)	49 (96%)	22 (100%)	0.35

^†^median (interquartile range)

^¶^The value for βD glucan is missing for one patient

HIV: human immunodeficiency virus, CMV: cytomegarlovirus, PaO_2_: partial pressure of oxygen in arterial blood, A-aDO_2_: alveolar-arterial oxygen difference, LDH: lactate dehydrogenase, CRP: C-reactive protein, PCP: pneumocystis pneumonia, cART: combination antiretroviral therapy.

The median duration of corticosteroid use was 13 days (IQR 9–21 days) and total dosage of corticosteroid was 405 mg (IQR 315–1133 mg) with prednisolone equivalent. Whereas 19 (26%) patients were treated with systemic corticosteroids for ≥21 days, 43 (59%) patients used corticosteroids for <15 days, 22 (30%) for <10 days, and 7 patients were not treated with systemic corticosteroids at all. The baseline A-aDO_2_ was ≥45 mm Hg in 51 (70%) patients and were thus categorized as the severe group, whereas 22 (30%) formed the moderate group (Table 1). The median duration of corticosteroid therapy was 15 days (IQR 11–24 days) for the severe group and 9 days (IQR 9–13 days) for the moderate group. The median total dosage of corticosteroids equivalent as prednisolone was 570 mg (IQR 345–4170 mg) for the severe group and 315 mg (IQR 184–361) for the moderate group. Among the 51 patients of the severe group, 18 (35%) were treated with corticosteroids for ≥21 days. On the other hand, only 2 (9%) patients of the moderate group were treated with corticosteroids for more than 14 days. The severe group had higher baseline LDH value compared with the moderate group (474 versus 379 U/L, p<0.001). However, the CD4 count and HIV load were not different between the two groups (CD4 count: 35 versus 41 /μL, p = 0.69; HIV load: 5.18 versus 5.23 log_10_/mL, respectively, p = 0.79). 26 (51%) of the severe group were with confirmed diagnosis with histological or cytological evidence, whereas 14 (64%) of the moderate group were with confirmed diagnosis, although the difference was not significant (p = 0.44). Some patients, particularly among those in the severe group, could not undergo bronchoscopy for histological evidence of PCP due to severe respiratory failures. Table 2 shows the baseline characteristics of the patients with severe PCP, after subdivision into the two groups based on the duration of corticosteroid therapy; those who required >14 days of corticosteroid therapy (the long corticosteroid group) and those who required ≤14 days (the short corticosteroid group). Patients in the long corticosteroid group had significantly lower CD4 counts (26 versus 54 /μL p = 0.049) and marginally higher LDH (478 versus 379 U/L p = 0.06) than those in the short corticosteroid group. The A-aDO_2_ and PaO_2_ values were not different between the two groups (A-aDO_2_: 71 versus 68 mmHg, p = 0.20; PaO_2_: 68 versus 69 mmHg, p = 0.24).

**Table 2:** Baseline characteristics of severe pneumocystis pneumonia patients according to the duration of corticosteroid therapy.

Variables at PCP diagnosis	Corticosteroids ≤14 days (n = 23)	Corticosteroids >14 days (n = 28)	P value
Age (years)^†^	37 (32–47)	42 (35–55)	0.22
Males, n (%)	29 (91%)	25 (89%)	0.81
CD4 count (/μL)^†^	54 (15–117)	26 (12–48)	0.049
HIV-1 load (log_10_/mL)^†^	5.15 (4.85–5.68)	5.23 (4.85–5.82)	0.61
Opportunistic infections other than PCP, n (%)	17 (70%)	21 (75%)	0.67
Cryptococcosis, n (%)	1 (4%)	1 (4%)	
Tuberculosis, n (%)	0	0	
CMV end-organ diseases, n (%)	0	0	
β-D glucan (pg/mL)^†^	240 (133–854)	221 (141–829)	0.84
PaO_2_ (mmHg)^†^	69 (55–116)	68 (48–82)	0.24
AaDO_2_ (mmHg)^†^	68 (53–101)	71 (56–139)	0.20
LDH (U/L)^†^	379 (327–460)	478 (426–663)	0.06
CRP (mg/dl)^†^	5.91 (1.31–11.0)	6.67 (2.86–10.07)	0.76
Total corticosteroid dosage (mg) (prednisolone equivalent)^†^	345(315–420)	1133 (572–4106)	0.08
Duration of corticosteroid therapy (days)^†^	10 (10–12)	23 (18–37)	<0.001

^†^median (interquartile range)

HIV: human immunodeficiency virus, CMV: cytomegarlovirus, PaO_2_: partial pressure of oxygen in arterial blood, A-aDO_2_: alveolar-arterial oxygen difference, LDH: lactate dehydrogenase, CRP: C-reactive protein, PCP: pneumocystis pneumonia.

With regard to prognosis of the study patients, among the moderate group two patients developed cryptococcosis and two developed tuberculosis within one year after the diagnosis of PCP. Among the severe group, one patient each developed cryptococcosis, cytomegalovirus retinitis, and cytomegalovirus colitis. All study patients were hospitalized and four patients out of 73 died with the mortality rate of 5%. All four patients who died were categorized into the severe disease and required more than 21 days of steroid, except for one patient who died on day 22, who discontinued steroid on day 19 due to his critical condition. Two (3%) patients were intubated and both of them died. One of them was intubated on day 14 due to aggravation of respiratory status and died on day 32. The other was intubated on day 12 due to worsening of respiratory failure and died on day 22. With regard to patients who died without intubation, one patient experienced multiple episodes of pneumothorax after admission (on day 19 and 59) and died on 62th hospital day because of respiratory failure. The other patient was diagnosed of cryptococcosis and cytomegalovirus colitis after diagnosis of PCP and the general condition of the patient gradually deteriorated and died on day 284. The prognosis of 7 patients who did not initiate steroid was favorable, as none died or intubated and were discharged.

## Discussion

This single-center study described the length of systemic corticosteroid therapy in PCP patients with HIV-1 infection who fulfilled the criteria of the American CDC guidelines for systemic corticosteroid therapy of 21-days. The median duration of corticosteroid therapy of the study patients was 13 days (IQR 9–21); corticosteroid therapy was discontinued in <15 days in 59% of the patients, and even within 9 days in 30% of the patients. Of the moderate group (A-aDO_2_ <45 mmHg), the median duration of corticosteroid therapy was only 9 days (IQR 9–13), and corticosteroid was discontinued within 14 days in 91% of the patients. On the other hand, patients of the severe group (A-aDO_2_ ≥45 mm Hg) required longer duration of corticosteroid therapy, as 55% required corticosteroids for >14 days and 31% required corticosteroids for >21 days. When we further categorized patients of the severe group into the two groups [long corticosteroid use (>14 days) and short corticosteroid use (≤14 days)], short corticosteroid therapy was associated with significantly higher CD4 count, and marginally associated with lower LDH value. The results of the present study might suggest that systemic corticosteroid therapy could be discontinued within 14 days in the majority of HIV-infected PCP with moderate-to-severe disease, rather than 21 days, and most (90% in this study) PCP with moderate severity could discontinue corticosteroid within 14 days. A large-scale prospective study, desirably randomized controlled trial that compares shorter versus longer courses of adjunctive corticosteroids in patients with moderate to severe disease, is warranted to confirm the results of the present study.

To our knowledge, this is the first study that investigated the duration of systemic corticosteroid therapy in moderate-to-severe PCP patients with HIV infection in the cART era under the observational setting. It is evident that short-term corticosteroid therapy is desirable in order to avoid various side effects of systemic steroids [8,11]. In this regard, one important finding of the present study is that systemic corticosteroid therapy for only 14 days was clinically effective in the majority of moderate-to-severe PCP and most moderate PCP patients.

A few randomized trials have demonstrated the benefits of corticosteroids during treatment of PCP patients with HIV infection, especially in patients with moderate-to-severe abnormalities in oxygen exchange at the time of presentation [12–14]. These trials were cited as evidence in the current guidelines, which recommend the 21-day of adjunctive corticosteroid use for HIV-1-infected moderate-to-severe PCP [4]. What is the underlying mechanism of the beneficial effects of corticosteroid therapy in PCP? Patients with PCP typically show clinical worsening after two to three days of therapy, presumably due to increased inflammation in response to dying organisms [15]. It is speculated that corticosteroid could alleviate this response and thus improved prognosis. In one study that failed to demonstrate the benefits of adjunctive corticosteroid, corticosteroid therapy commenced only when the criteria for respiratory deterioration were met during PCP treatment, and this was not necessarily at the beginning of the treatment [15]. This finding might support our hypothesis that starting corticosteroid therapy at the beginning of treatment of PCP patients with HIV-1 infection is particularly important, and that continuation of such treatment for 21 days is not necessary for all patients with moderate-to-severe PCP. Furthermore, two small randomized trials showed that 7 to 10 days of adjunctive corticosteroid started within 24–72 hours of the first dose of antimicrobial therapy reduced short-term mortality in PCP patients with HIV infection [13,16]. Based on the findings of the above previous studies, it is important to use corticosteroids as early as possible and certainly within 72 hours after the start of treatment for PCP therapy.

Apart from the treatment, it is also noteworthy that 97% of the patients with moderate to severe PCP in this study were treatment-naïve for HIV infection, suggesting that the admitting physician needs to maintain a high index of clinical suspicion for PCP at the time of presentation, especially if there is no previously known history of HIV infection.

The present study has several limitations. First, the number of enrolled patients was small, and some results of the present study, such as comparison of patients with severe PCP who were treated with corticosteroids for ≤14 days and >14 days, need to be interpreted with caution. Second, the study was retrospective in nature and the duration of corticosteroid was at the discretion of the treating physicians. Also, it is important to note that some patients with severe PCP required corticosteroid therapy for ≥21 days (26% of the study patients were actually treated with corticosteroids for ≥21 days), although the corticosteroid was discontinued within 14 days in the majority of moderate-to-severe PCP and most moderate PCP. Third, the study patients included clinically-diagnosed (probable) PCP cases without histological or cytological evidence. However, all probable cases underwent chest CT and serum β-D glucan, both of which have high diagnostic value for PCP [2,17], presented with symptoms compatible with PCP, and responded clinically after specific anti-PCP treatment, such as sulfamethoxazole/trimethoprim, intravenous pentamidine, and atovaquone. Other agents, such as broad-spectrum antimicrobials, were not used for any probable cases. Furthermore, many major papers, including ACTG clinical trials, in the field of HIV-infected PCP included both histologically or cytologically confirmed cases and clinically-diagnosed (probable) cases [10]. It is also noteworthy that, in clinical practice, some PCP cases with severe respiratory failure cannot undergo bronchoalveolar lavage, and if such cases were excluded from the analysis, the results would not reflect the actual data gained from the observational settings.

In conclusion, the present study suggested that adjunctive corticosteroid therapy for only 14 days or less was clinically effective in 60% of HIV patients with moderate-to-severe PCP and 90% of moderate cases who fulfilled the criteria for use of corticosteroids for 21 days. Because short-term corticosteroid therapy is desirable in order to avoid various side effects of systemic corticosteroid, it is important to note that the majority of such patients can discontinue corticosteroid within 14 days.

## References

[1] Bonnet F, Lewden C, May T, Heripret L, Jougla E, Bevilacqua S, et al (2005) Opportunistic infections as causes of death in HIV-infected patients in the HAART era in France. Scand J Infect Dis 37: 482–487. 16089023 10.1080/00365540510035328

[2] Thomas CF Jr, Limper AH (2004) Pneumocystis pneumonia. N Engl J Med 350: 2487–2498. 15190141 10.1056/NEJMra032588

[3] Wolff AJ, O'Donnell AE (2003) HIV-related pulmonary infections: a review of the recent literature. Curr Opin Pulm Med 9: 210–214. 12682566 10.1097/00063198-200305000-00009

[4] Panel on Opportunistic Infections in HIV-Infected Adults and Adolescents. Guidelines for the prevention and treatment of opportunistic infections in HIV-infected adults and adolescents: recommendations from the Centers for Disease Control and Prevention, the National Institutes of Health, and the HIV Medicine Association of the Infectious Diseases Society of America. Available: http://aidsinfo.nih.gov/contentfiles/lvguidelines/adult_oi.pdf. Accessed 2015 March 1.

[5] Stuck AE, Minder CE, Frey FJ (1989) Risk of infectious complications in patients taking glucocorticosteroids. Rev Infect Dis 11: 954–963. 2690289 10.1093/clinids/11.6.954

[6] Ginzler E, Diamond H, Kaplan D, Weiner M, Schlesinger M, Seleznick M (1978) Computer analysis of factors influencing frequency of infection in systemic lupus erythematosus. Arthritis Rheum 21: 37–44. 414759 10.1002/art.1780210107

[7] Wolfe F, Caplan L, Michaud K (2006) Treatment for rheumatoid arthritis and the risk of hospitalization for pneumonia: associations with prednisone, disease-modifying antirheumatic drugs, and anti-tumor necrosis factor therapy. Arthritis Rheum 54: 628–634. 16447241 10.1002/art.21568

[8] Ko JH, Peck KR, Lee WJ, Lee JY, Cho SY, Ha YE, et al (2015) Clinical presentation and risk factors for cytomegalovirus colitis in immunocompetent adult patients. Clin Infect Dis 60: e20–26. 10.1093/cid/ciu969 25452594

[9] Nishijima T, Komatsu H, Gatanaga H, Aoki T, Watanabe K, Kinai E, et al (2011) Impact of small body weight on tenofovir-associated renal dysfunction in HIV-infected patients: a retrospective cohort study of Japanese patients. PLoS One 6: e22661 10.1371/journal.pone.0022661 21799928 PMC3143186

[10] Sax PE, Komarow L, Finkelman MA, Grant PM, Andersen J, Scully E, et al (2011) Blood (1->3)-beta-D-glucan as a diagnostic test for HIV-related Pneumocystis jirovecii pneumonia. Clin Infect Dis 53: 197–202. 10.1093/cid/cir335 21690628 PMC3165964

[11] Buchman AL (2001) Side effects of corticosteroid therapy. J Clin Gastroenterol 33: 289–294. 11588541 10.1097/00004836-200110000-00006

[12] Montaner JS, Lawson LM, Levitt N, Belzberg A, Schechter MT, Ruedy J (1990) Corticosteroids prevent early deterioration in patients with moderately severe Pneumocystis carinii pneumonia and the acquired immunodeficiency syndrome (AIDS). Ann Intern Med 113: 14–20. 2190515 10.7326/0003-4819-113-1-14

[13] Nielsen TL, Eeftinck Schattenkerk JK, Jensen BN, Lundgren JD, Gerstoft J, van Steenwijk RP, et al (1992) Adjunctive corticosteroid therapy for Pneumocystis carinii pneumonia in AIDS: a randomized European multicenter open label study. J Acquir Immune Defic Syndr 5: 726–731. 1613673

[14] Bozzette SA, Sattler FR, Chiu J, Wu AW, Gluckstein D, Kemper C, et al (1990) A controlled trial of early adjunctive treatment with corticosteroids for Pneumocystis carinii pneumonia in the acquired immunodeficiency syndrome. California Collaborative Treatment Group. N Engl J Med 323: 1451–1457. 2233917 10.1056/NEJM199011223232104

[15] The National Institutes of Health-University of California Expert Panel for Corticosteroids as Adjunctive Therapy for Pneumocystis Pneumonia. (1990) Consensus statement on the use of corticosteroids as adjunctive therapy for pneumocystis pneumonia in the acquired immunodeficiency syndrome. N Engl J Med 323: 1500–1504. 2136587 10.1056/NEJM199011223232131

[16] Gagnon S, Boota AM, Fischl MA, Baier H, Kirksey OW, La Voie L (1990) Corticosteroids as adjunctive therapy for severe Pneumocystis carinii pneumonia in the acquired immunodeficiency syndrome. A double-blind, placebo-controlled trial. N Engl J Med 323: 1444–1450. 2233916 10.1056/NEJM199011223232103

[17] Watanabe T, Yasuoka A, Tanuma J, Yazaki H, Honda H, Tsukada K, et al (2009) Serum (1—>3) beta-D-glucan as a noninvasive adjunct marker for the diagnosis of Pneumocystis pneumonia in patients with AIDS. Clin Infect Dis 49: 1128–1131. 10.1086/605579 19725788

